# Improved Glucose Metabolism In Vitro and In Vivo by an Allosteric Monoclonal Antibody That Increases Insulin Receptor Binding Affinity

**DOI:** 10.1371/journal.pone.0088684

**Published:** 2014-02-12

**Authors:** John A. Corbin, Vinay Bhaskar, Ira D. Goldfine, Daniel H. Bedinger, Angela Lau, Kristen Michelson, Lisa M. Gross, Betty A. Maddux, Hua F. Kuan, Catarina Tran, Llewelyn Lao, Masahisa Handa, Susan R. Watson, Ajay J. Narasimha, Shirley Zhu, Raphael Levy, Lynn Webster, Sujeewa D. Wijesuriya, Naichi Liu, Xiaorong Wu, David Chemla-Vogel, Steve R. Lee, Steve Wong, Diane Wilcock, Mark L. White

**Affiliations:** 1 Department of Preclinical Research, XOMA Corporation, Berkeley, California, United States of America; 2 Department of Medicine, University of California San Francisco, San Francisco, California, United States of America; University of Bremen, Germany

## Abstract

Previously we reported studies of XMetA, an agonist antibody to the insulin receptor (INSR). We have now utilized phage display to identify XMetS, a novel monoclonal antibody to the INSR. Biophysical studies demonstrated that XMetS bound to the human and mouse INSR with picomolar affinity. Unlike monoclonal antibody XMetA, XMetS alone had little or no agonist effect on the INSR. However, XMetS was a strong positive allosteric modulator of the INSR that increased the binding affinity for insulin nearly 20-fold. XMetS potentiated insulin-stimulated INSR signaling ∼15-fold or greater including; autophosphorylation of the INSR, phosphorylation of Akt, a major enzyme in the metabolic pathway, and phosphorylation of Erk, a major enzyme in the growth pathway. The enhanced signaling effects of XMetS were more pronounced with Akt than with Erk. In cultured cells, XMetS also enhanced insulin-stimulated glucose transport. In contrast to its effects on the INSR, XMetS did not potentiate IGF-1 activation of the IGF-1 receptor. We studied the effect of XMetS treatment in two mouse models of insulin resistance and diabetes. The first was the diet induced obesity mouse, a hyperinsulinemic, insulin resistant animal, and the second was the multi-low dose streptozotocin/high-fat diet mouse, an insulinopenic, insulin resistant animal. In both models, XMetS normalized fasting blood glucose levels and glucose tolerance. In concert with its ability to potentiate insulin action at the INSR, XMetS reduced insulin and C-peptide levels in both mouse models. XMetS improved the response to exogenous insulin without causing hypoglycemia. These data indicate that an allosteric monoclonal antibody can be generated that markedly enhances the binding affinity of insulin to the INSR. These data also suggest that an INSR monoclonal antibody with these characteristics may have the potential to both improve glucose metabolism in insulinopenic type 2 diabetes mellitus and correct compensatory hyperinsulinism in insulin resistant conditions.

## Introduction

It has been proposed that receptor antibodies may represent a novel class of therapeutics for regulating glucose metabolism in type 2 diabetes mellitus (T2DM) [1]. The insulin receptor (INSR) is a central node for glycemic control in cells of the major metabolic insulin responsive tissues and therefore, is a key target for antibodies that could either mimic or potentiate insulin action in diabetes [2]. Spontaneously occurring human INSR autoantibodies, and mouse monoclonal antibodies generated to the human INSR have been investigated [3]–[13]. In humans, autoantibodies to the INSR typically cause severe insulin resistance [6], [7], [10]. Very rarely, INSR autoantibodies bind to and stimulate the INSR resulting in hypoglycemia [6], [8]. In addition, monoclonal antibodies to the INSR produced in mice have been used to characterize this receptor [3]–[5], [14]. Some of these monoclonal antibodies have been shown to mimic insulin action in vitro, but they have not been tested in animal models of diabetes.

Many of the aforementioned antibodies to the INSR inhibit insulin binding to the orthosteric site (insulin binding site). In addition, antibodies that bind to allosteric sites (not the orthosteric site) of receptors can also impact cell signaling [15]–[17]. Recently, we reported the discovery and characterization of XMetA, an allosteric antibody to the INSR that was a direct agonist [18], [19]. XMetA had had no effect on the binding of insulin to the INSR; however it stimulated INSR signaling in cultured cells and reduced hyperglycemia in mouse models of diabetes. In addition to being agonists, allosteric antibodies could also act as positive allosteric modulators of the INSR by enhancing insulin binding affinity and increasing metabolic signaling, without directly activating the INSR. In the present study we describe the discovery and characterization of one such positive allosteric modulator of the INSR, XMetS. In cultured cells, XMetS markedly enhanced insulin binding affinity leading to potentiation of insulin-stimulated INSR signaling resulting in enhanced glucose transport. Moreover, XMetS reduced hyperinsulinemia and hyperglycemia in two mouse models of insulin resistance and diabetes.

## Research Design and Methods

### XMetS Discovery

The extracellular domain of the human INSR (hINSR) (R&D Systems, MN) was biotinylated (Sulfo-NHS-LC-Biotin, Pierce, Rockford, IL) and incubated with a saturating concentration (10 µM) of human insulin (hINS; Sigma-Aldrich, St. Louis, MO) to complex the INSR with insulin. These complexes were conjugated to streptavidin-coated magnetic beads (Dynabeads® M-280, Invitrogen Dynal AS, Oslo, Norway) to generate the panning reagent. All subsequent steps were carried out in the presence of 10 µM human insulin to maintain biotinylated hINSR that was complexed to hINS (biotin-hINSR/hINS).

Two naïve human antibody phage display libraries (XOMA Corporation, Berkeley, CA) were panned employing standard methods [20], [21]. Prior to panning, phage were deselected against unconjugated streptavidin-coated magnetic beads to remove nonspecific phage antibodies. Deselected phage were then incubated with biotin-hINSR/hINS streptavidin beads. hINSR/hINS streptavidin bead-bound phage were eluted and used to infect TG1 bacterial cells (Stratagene, La Jolla, CA). Phage were then rescued with helper phage M13KO7 (New England Biolabs, MA). Individual colonies were picked and grown in 96-well plates, and were then used to generate bacterial periplasmic extracts according to standard methods [20]. The binding properties of monovalent Fab and scFv anti-INSR antibodies from lysates were screened by FACS (fluorescence-activated cell sorting).

For the majority of the subsequent functional studies shown herein, we employed CHO cells transfected with the B isoform of either the human INSR (CHO-hINSR) or mouse INSR (CHO-mINSR). The B isoform of the INSR was employed because it is the predominant isoform of the INSR in adult metabolic insulin responsive tissues [22], [23]. As determined by FACS [24], both these human and mouse INSR-transfected cell lines had approximately 250,000 surface receptors per cell compared to untransfected CHO cells (parental CHO cells), which had less than 5,000 surface INSR per cell. For comparative studies of XMetS and insulin binding, CHO cells were also transfected with the A isoform of the hINSR and were selected to have approximately 250,000 surface receptors per cell. Under the conditions employed for the subsequent cellular studies, the surface receptor levels did not change. INSR molar concentrations in the assays were calculated based on the cell surface receptor content, number of cells utilized, and the volume of the reaction. For comparative studies of receptor autophosphorylation, CHO cells transfected with the human insulin-like growth factor-1 receptors (CHO-hIGF-1R) were studied that had approximately 300,000 surface receptors per cell.

Screening studies using both CHO-hINSR and CHO-mINSR cells identified XMetS, an antibody that preferentially bound to the insulin occupied INSR (but not insulin alone), indicating the potential for positive allosteric modulation activity [25]. This clone was reformatted into a fully human divalent IgG2 monoclonal antibody that was employed in all studies described herein unless indicated otherwise. A monovalent Fab version of XMetS was also generated and tested.

### XMetS Binding as Assessed by FACS

For FACS, CHO-hINSR cells (2×10^6^/ml) were washed and resuspended in phosphate buffered saline (PBS) with 0.5% fatty acid-free bovine serum albumin and 0.1% sodium azide (FACS buffer; Invitrogen, Carlsbad, CA). Cells were preincubated for 10 minutes at 4°C in the presence of 100 nM human insulin (Sigma-Aldrich). Next, increasing concentrations of XMetS were added to the cell suspension and incubated at 15°C for 120 minutes. Cells were then washed and resuspended in Alexa Fluor® 647-conjugated goat anti-human IgG (1∶200; Invitrogen, Carlsbad, CA). The cells were incubated for 30 minutes at 4°C, washed twice and analyzed on a FACScan™ flow cytometer (Becton Dickinson, San Jose, CA).

### XMetS and Insulin Binding Assessed by Kinetic Exclusion Assay (KinExA™)

In order to measure the effect of insulin on the binding affinity of XMetS for both isoforms of the hINSR, and the B isoform of the mINSR, we employed equilibrium assays under conditions where there was either no insulin present or where saturating insulin concentrations (>100 nM) were present. XMetS (50 pM) was incubated for 18 hours on a rotator at 5°C in PBS with 0.25% bovine serum albumin and 0.1% sodium azide with increasing concentrations of CHO cells (max 2×10^7^/ml) expressing either the hINSR or mINSR. Following this incubation, the viability of all cells was greater than 85% as measured by trypan blue exclusion. Cells were pelleted by centrifugation and the amount of free XMetS remaining in solution was measured by immunofluorescence using a KinExA™ instrument (Sāpidyne Instruments, Boise, Idaho) [26]. Briefly, polymethylmethacrylate (PMMA) beads (Sāpidyne Instruments) were coated with 65 µg/mL goat anti-human-IgG antibody (Jackson Immuno Research, West Grove, PA) and captured human antibody was detected with an anti-human IgG-PE labeled antibody (Jackson Immuno Research) diluted 1∶1000. XMetS concentration data were curve-fit using KinExA™ software (standard affinity curve fit model) [27] to yield an estimate of the relative change in XMetS binding affinity as expressed in terms of equilibrium dissociation (K_D_) values.

In order to measure the effect of XMetS on the binding affinity of insulin for both isoforms of the hINSR, and the B isoform of the mINSR, we employed equilibrium assays under conditions where there was either no XMetS or where saturating XMetS concentrations (>30 nM) were present. Human insulin (50 pM, Sigma-Aldrich) and either XMetS or an anti-keyhole limpet hemocyanin IgG2 isotype control antibody (>30 nM) were incubated for 18 hours on a rotator at 5°C in PBS with 0.25% bovine serum albumin and 0.1% sodium azide with increasing concentrations of either CHO-hINSR or CHO-mINSR cells. Cells were pelleted by centrifugation and the amount of free insulin in solution was measured by immunofluorescence using a KinExA™ instrument [26]. Briefly, PMMA beads (Sapidyne Instruments) were coated with 65 µg/mL D6C4 anti-insulin monoclonal Ab (Fitzgerald Industries) and captured insulin was detected with 0.15 µg/mL biotin labeled D3E7 anti-insulin monoclonal antibody (Fitzgerald Industries, Acton, MA) mixed with 1 µg/mL streptavidin-phycoerythrin (Jackson Immuno Research). Insulin concentration data were curve-fit using KinExA™ software [27] (standard affinity curve fit model) to yield an estimate of the relative change in insulin binding affinity as expressed in terms of equilibrium dissociation (K_D_) values.

The impact of XMetS, on the kinetics of insulin binding to the hINSR was assessed with time course studies under conditions where the XMetS concentration was saturating (>30 nM). Either XMetS or control antibody was preincubated for 30 minutes at 5°C with CHO-hINSR cells expressing the B isoform of the hINSR (2×10^7^/ml). Next, human insulin (200 pM) was added and cells were incubated for up to 80 minutes at 5°C. At each time point of the incubation period, an aliquot of cells was pelleted by centrifugation. The concentration of free insulin remaining in the supernatant was measured using an electrochemiluminescent assay (Meso Scale Discovery, Gaithersburg, MD) and bound insulin was calculated. Briefly, MSD (Meso Scale Discovery) plates were coated 10 µg/mL D6C4 anti-insulin monoclonal antibody (Fitzgerald Industries) and captured insulin was detected with 0.3 µg/mL biotin labeled D3E7 anti-insulin monoclonal antibody (Fitzgerald Industries) mixed with 1.3 µg/mL streptavidin-europium (Meso Scale Discovery). Insulin concentration data and equilibrium dissociation (K_D_) values determined by the aforementioned equilibrium KinExA™ assays were used to curve-fit the data and calculate the on-rate and off-rate of insulin by employing KinExA™ software (direct kinetics curve fit model).

### The Effect of XMetS on Insulin Signaling in Cultured Cells

For studies of receptor autophosphorylation, CHO cells expressing either the hINSR or the hIGF-1R were preincubated in serum-free culture medium with XMetS (33 nM) for 30 minutes at 37°C followed by a 10-minute incubation with increasing concentrations of the cognate ligand (either insulin or IGF-1). Quantitation of tyrosine phosphorylated INSR and IGF-1R were determined by ELISA (Millipore, Billerica, MA) [28]. Receptor autophosphorylation data were curve-fit using GraphPad Prism™ software (sigmoidal dose-response, variable slope) to generate EC_50_ values.

For studies of Akt phosphorylation, CHO cells expressing the hINSR B isoform were preincubated in serum-free culture medium with either XMetS (14 nM for the IgG2 and 140 nM for the Fab) or 70 nM control antibody for 10 minutes at 37°C followed by a 10-minute incubation with increasing concentrations of insulin. Total Akt and Akt phosphorylated at Ser473 were quantified using an electrochemiluminescent assay (Meso Scale Discovery). Akt phosphorylation data were curve-fit using GraphPad Prism™ software (sigmoidal dose-response, variable slope) to generate EC_50_ values.

In order to quantitate the positive allosteric modulatory effects of XMetS on insulin-stimulated Akt activation by the INSR, a Schild analysis was conducted [25], [29], [30]. CHO cells expressing the hINSR were preincubated in serum-free culture medium with XMetS concentrations ranging from 0–50 nM for 10 minutes at 37°C followed by a 10 minute incubation with increasing concentrations of insulin. Total Akt and Akt phosphorylated at Ser473 were quantified as above, and the data were curve-fit using GraphPad Prism™ software (allosteric EC50 shift) to generate the parameters of INSR allosteric modulation by XMetS.

For studies of Erk1/2 phosphorylation, CHO cells expressing the hINSR B isoform were preincubated in serum-free culture medium with 33 nM XMetS for 10 minutes at 37°C followed by a 10 minute incubation with increasing concentrations of either insulin. Total Erk1/2 and Erk1/2 phosphorylated at Thr202/Tyr204 were measured using an electrochemiluminescent assay (Meso Scale Discovery). Erk1/2 phosphorylation data were curve-fit using GraphPad Prism™ software (sigmoidal dose-response, variable slope) to generate EC_50_ values.

To measure 2-deoxy-glucose uptake, rat L6 muscle cells expressing both hINSR B isoform and human GLUT-4 [31] were preincubated in serum-free medium for 60 minutes at 37°C with either 33 nM XMetS or control antibody. Next, cells were incubated with increasing concentrations of insulin for 10 minutes. [^3^H]-2-deoxy-D-glucose was then added for 20 minutes and its uptake measured [14], [31]. Glucose uptake data were curve-fit using GraphPad Prism™ software (sigmoidal dose-response, variable slope) to generate EC_50_ values.

MCF-7 human breast cancer cells (ATCC, Manassas, VA) were cultured in Dulbecco’s Modified Eagles Medium (DMEM) containing glucose at 4.5 g/L supplemented with 10% FBS and 2 mM glutamine (Invitrogen, Carlsbad, CA) for normal maintenance. For the proliferation assay, cells (1×10^4^) were seeded in 96 well white opaque microtiter plates and allowed to reattach. After 24 hrs, cells were washed twice with pre-warmed PBS. They were then incubated in DMEM (without phenol red) containing glucose at 1 g/L supplemented with 0.1% FBS and 2 mM glutamine (Invitrogen, Carlsbad, CA) for another 24 hrs. Next, either 33 nM XMetS or control antibody was added to the cells along with varying concentrations of either insulin, IGF-1 or IGF-2 (Sigma-Aldrich, St. Louis, MO). Cells were incubated at 37°C for 48 hrs and cell proliferation was measured using the CellTiter-Glo® Luminescent Cell Viability Assay (Promega, Madison, WI). Proliferation data were curve-fit using Prism™ software (sigmoidal dose-response) to generate EC_50_ values. Saos-2 osteosarcoma cells (ATCC, Manassas, VA) were treated in a similar manner except that they were incubated for 72 hrs prior to measurement of cell proliferation.

### Ethics Statement

Animal experiments were approved by the XOMA Corporation Institutional Animal Care and Use Committee (IACUC) and performed in accordance with IACUC guidelines. All animals were maintained in a pathogen-free environment and allowed free access to food and water.

### Mouse Models of Insulin Resistance and Diabetes

In the diet-induced obesity model [32] (18–20 weeks of age), C57BL/6 mice (The Jackson Laboratory, Sacramento, CA) were fed a high fat diet (65 kcal% fat) for 12–14 weeks. Mice were then randomized into two groups (*n* = 10). Mice were treated twice weekly, by intraperitoneal (IP) injection, with either XMetS or the anti-keyhole limpet hemocyanin IgG2 control antibody (10 mg/kg). Age-matched, lean C57BL/6 mice were fed a standard chow diet (Harlan, Indianapolis, IN) and treated twice weekly with the control antibody (10 mg/kg). A glucose tolerance test was performed after one week of antibody administration. For the glucose tolerance test, mice were fasted overnight for 14 hours followed by a glucose challenge (1 g/kg; Mediatech, Manassas, VA) by IP injection. Whole venous blood was obtained from the tail vein at 0, 15, 30, 60 90, and 120 minutes following challenge and evaluated for blood glucose. For all mouse studies, blood glucose was measured using the AlphaTRAK® Blood Glucose Monitoring System (Abbott, Chicago, IL).

In the multi-low dose streptozotocin/high-fat diet model [33], six-week old male ICR mice were fed a 40 kcal% fat, 35 kcal% sucrose diet (Research Diets, New Brunswick, NJ) for 4 weeks. Streptozotocin (Sigma, St. Louis, MO) was injected IP at 40 mg/kg for 5 consecutive days during the third week of high fat diet feeding. After an additional week, mice were randomized into two groups (*n* = 8). They were then treated twice weekly by IP injection with either XMetS or control antibody (10 mg/kg). Non-diabetic ICR mice were fed a standard chow diet (Harlan, Indianapolis, IN) and treated twice weekly with the control antibody (10 mg/kg). Fasting blood glucose was evaluated weekly following a 14-hour overnight fast. An IP glucose tolerance test (1 g/kg glucose) was performed after 3 weeks of antibody administration, following a 14-hour overnight fast. After five weeks of antibody administration, insulin tolerance tests were carried out following a 4-hour fast by administering insulin (0.75 U/kg; Roche Diagnostics, Indianapolis, IN) intraperitoneally and measuring venous blood for glucose at 0, 30, 60, 90 and 120 minutes after insulin challenge.

At the conclusion of the above studies (multi-low dose streptozotocin/high-fat diet = 6 weeks; diet induced obesity = 4 weeks) mice were sacrificed and terminal plasma was collected by cardiac puncture following a 14-hour overnight fast. Fasting values for plasma glucose, beta-hydroxybutyrate (multi-low dose streptozotocin/high-fat diet only), total cholesterol and HDL cholesterol were measured by standard colorimetric methods (Wako Chemicals, Richmond, VA). Insulin and C-peptide were measured by ELISA (Alpco Diagnostics, Salem NH). Statistical analyses were carried out using a two-tailed student’s unpaired t test.

## Results

### I. Studies in vitro with Cultured Cells

#### XMetS binds to the INSR

**Figure 1:**
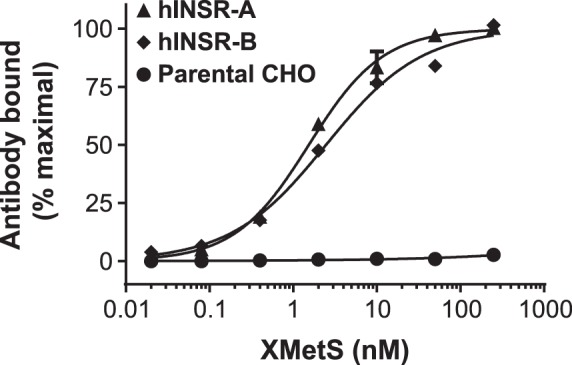
XMetS binds to the A and B isoforms of the INSR with high affinity. CHO cells expressing either the B or A isoform of the hINSR, and parental CHO cells were preincubated for 10 minutes at 4°C in the presence of 100 nM insulin followed by a 120 minute incubation at 15°C with increasing concentrations of XMetS. XMetS binding to the INSR was measured by FACS. Mean of duplicate determinations are shown.

Allosteric modulating antibodies that potentiated the binding of insulin to the INSR were identified by panning naïve human antibody phage display libraries against the recombinant extracellular domain of the INSR which was complexed to insulin. Antibodies that preferentially bound to the INSR in the presence of insulin were then identified. This panning and screening approach was designed to specifically select for positive allosteric modulating antibodies because of the reciprocal relationship between a positive allosteric modulator and the ligand (i.e. the modulator enhances the binding affinity of the ligand, and in turn, the ligand enhances the binding affinity of the modulator) [25]. Using FACS, we qualitatively assessed the binding of XMetS to the INSR B and A isoforms (CHO-hINSR B and CHO-hINSR A cells) in cultured CHO cells expressing high levels of both isoforms. XMetS bound to both INSR isoforms in a similar manner (**Figure 1**). XMetS also bound similarly to the B isoform of the mINSR (data not shown). XMetS did not to parental CHO cells (**Figure 1**).

#### Quantitative studies defining XMetS as a positive allosteric modulator of the human and mouse INSR

**Figure 2:**
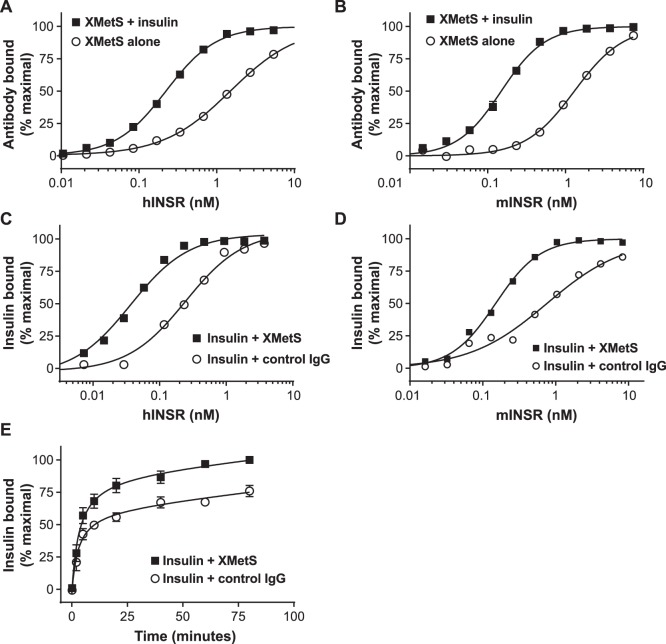
Insulin increases XMetS binding affinity to the INSR and XMetS increases insulin binding affinity to the INSR. A and **B** (*XMetS binding to the INSR*). 50 pM XMetS was incubated with increasing concentrations of either (**A**) CHO-hINSR cells or (**B**) CHO-mINSR for 18 hours at 5°C in the presence and absence of insulin. Free antibody concentrations were quantified by immunofluorescence (KinExA™), and this value was used to calculate bound XMetS. Mean of duplicate determinations are shown. **C** and **D** (*Insulin binding to the INSR*). 50 pM insulin was incubated with increasing concentrations of either (**C**) CHO-hINSR cells or (**D**) CHO-mINSR cells for 18 hours at 5°C in the presence of either XMetS or control antibody. Free insulin was quantified by immunofluorescence (KinExA™) and this value was used to calculate bound insulin. Mean of duplicate determinations are shown. **E**. Human insulin (200 pM) was incubated with CHO-hINSR cells for up to 80 minutes at 5°C with either XMetS or control antibody. At each time point, cells were pelleted by centrifugation. The concentration of free insulin in the supernatant was measured by immunofluorescence and bound insulin was then calculated. Mean ± SD from three separate experiments are shown.

Kinetic exclusion assays (KinExA™) were employed to quantitatively assess the reciprocal binding interactions between XMetS and insulin to the hINSR. First, the impact of insulin on the binding affinity of XMetS to the INSR was measured (**Figures 2A and 2B**). XMetS alone bound to CHO-hINSR cells expressing the B isoform of the receptor with an equilibrium dissociation constant (K_D_) of 3.0 nM (95% confidence interval (CI): 2.5 to 3.2 nM), but bound only weakly to parental CHO cells (data not shown). XMetS binding affinity was enhanced 16-fold (K_D_ = 190 pM, 95% CI: 140 to 250 pM) in the presence of insulin (**Figure 2A**). Similar results were obtained with the A isoform of the INSR; XMetS alone bound to CHO-hINSR cells expressing the A isoform of the receptor with an equilibrium dissociation constant (K_D_) of 4.0 nM (95% confidence interval (CI): 2.0 to 5.1 nM), and in the presence of insulin XMetS bound with a K_D_ of 139 pM (95% CI: 50 to 290 pM) (data not shown). The interaction of XMetS with the mINSR was also investigated. XMetS bound to CHO-mINSR cells with a K_D_ of 520 pM (95% CI: 197 pM to 1.1 nM) in the absence of insulin. As with CHO-hINSR B isoform cells, the binding affinity of XMetS to CHO-mINSR cells increased 16-fold in the presence of insulin (K_D_ = 33 pM, 95% CI: 10.4 to 66 pM) (**Figure 2B**).

Second, the impact of XMetS on the binding affinity of insulin to the INSR (**Figures 2C and 2D**) was assessed. In the absence of XMetS, insulin bound to CHO-hINSR cells expressing the B isoform of the receptor with a K_D_ of 270 pM (95% CI: 142 to 434 pM) (**Figure 2C**). In the presence of XMetS, binding of insulin was enhanced 18-fold (K_D_ of 15 pM, 95% CI: 6 to 23 pM). Similar results were obtained with the A isoform of the INSR; in the absence of XMetS, insulin bound to CHO-hINSR cells expressing the A isoform of the receptor with a K_D_ of 536 pM (95% CI: 27 to 920 pM), and in the presence of XMetS insulin bound with a K_D_ of 15 pM, 95% CI: 6 to 23 pM) (data not shown). In the absence of XMetS, insulin bound to CHO-mINSR cells with a K_D_ of 650 pM) 95% CI: 300 to 930 pM) (**Figure 2D**). In the presence of XMetS, binding of insulin to mINSR was enhanced 11-fold to 57 pM (95% CI: 17 to 124 pM). These data indicated that XMetS was a positive allosteric modulator of both the hINSR and mINSR that markedly enhanced insulin binding affinity. Moreover, the reciprocal nature of the binding interactions between XMetS and insulin further demonstrated that XMetS was a positive allosteric modulator [25] of the INSR. Because XMetS had similar effects on the A and B isoforms of the hINSR, as well as the mINSR, all subsequent in vitro experiments were carried out with the B isoform of the hINSR.

Time course studies were conducted to elucidate the kinetic parameters involved in the enhancement of insulin binding to the INSR by XMetS (**Figure 2E**). XMetS did not significantly alter the observed association rate of insulin binding to the CHO-hINSR cells. Based on the equilibrium K_D_ parameters determined above and these time course data, the calculated association rates [27] for insulin binding to the INSR in the presence or absence of XMetS were unchanged (8.5×10^5^ M^−1^, sec^−1^, 95% CI: 4.7×10^5^ to 1.4×10^6^ M^−1^, sec^−1^ vs. 9.5 x 10^5 ^M^−1^, sec^−1^, 95% CI: 6.2×10^5^ to 1.4×10^6^ M^−1^, sec^−1^). In contrast, the calculated dissociation rates in the presence or absence of XMetS were markedly different (2.2±1.7×10^−4 ^sec^−1^, and 1.4±0.7×10^−5^ sec^−1^ respectively). These data indicate that the positive modulation of insulin affinity by XMetS primarily resulted from a reduction of the dissociation rate between insulin and the INSR.

#### The effect of XMetS on insulin-mediated INSR autophosphorylation

**Figure 3:**
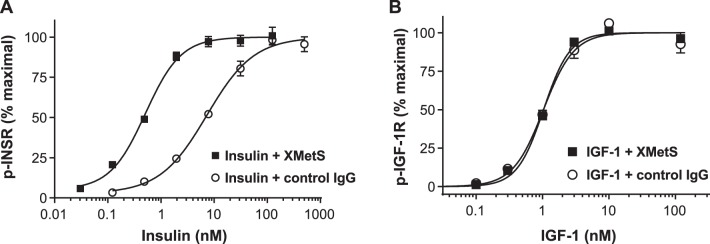
XMetS enhances insulin-dependent INSR autophosphorylation, but does not potentiate IGF-1-dependent IGF-1R autophosphorylation. **A.** CHO-hINSR cells were preincubated for 30 minutes at 37°C with either XMetS or control antibody, then incubated for 10 minutes with increasing concentrations of insulin. **B.** CHO-IGF-1R cells were preincubated for 30 minutes at 37°C with either XMetS or control antibody, then incubated for 10 minutes with increasing concentrations of IGF-1. Autophosphorylation was measured by ELISA. Mean ± SD from triplicate determinations are shown.

The binding of insulin to the INSR induces tyrosine autophosphorylation, the major indicator of receptor activation [2]. Similar to its enhancement of insulin binding affinity, XMetS induced a 14-fold increase in INSR autophosphorylation in response to insulin (**Figure 3A**), indicating that it was a positive allosteric modulator of both insulin binding and INSR activation. The EC_50_ for INSR autophosphorylation in the absence of XMetS was 7.3 nM (95% CI: 5.6 to 8.9 nM), but fell to 0.51 (95% CI: 0.44 to 0.6 nM) in the presence of XMetS. XMetS alone had no direct agonist effect on INSR autophosphorylation. The receptor for IGF-1 (IGF-1R) is closely related to the INSR [34]. In order to examine the receptor specificity of XMetS, we compared the effects of XMetS on IGF-1-mediated activation of the IGF-1R expressed in CHO cells (**Figure 3B**). In contrast to the sensitizing effect of XMetS on insulin stimulation of the INSR, the antibody had no effect on enhancing IGF-1-mediated IGF-1R autophosphorylation.

#### The effect of XMetS on insulin-mediated Akt and Erk activation in cells expressing the hINSR

**Figure 4:**
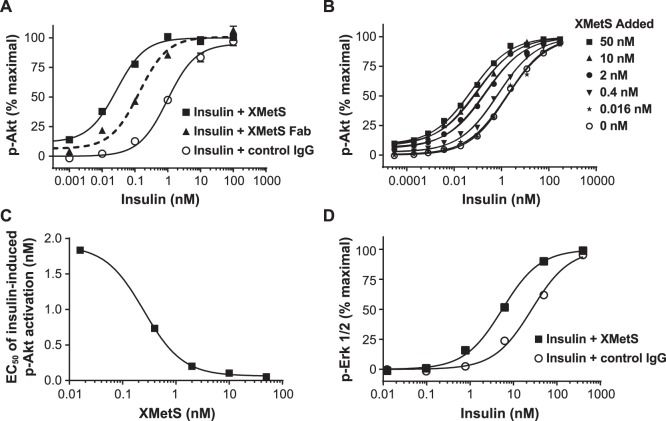
XMetS potentiates insulin-dependent Akt and Erk phosphorylation. **A.** CHO-hINSR cells were preincubated for 10 minutes at 37°C with either the IgG2 form of XMetS, the Fab form of XMetS, or control antibody followed by a 10 minute incubation with increasing concentrations of insulin. Phosphorylation of Akt was then determined. **B**. CHO-hINSR cells were preincubated for 10 minutes at 37°C with a wide range of XMetS concentrations followed by a 10 minute incubation with increasing concentrations of insulin. Schild analysis [30] of these data was carried out using a curve fit algorithm (Prism, allosteric EC_50_ shift). **C.** EC_50_ values were determined from each curve fit (of the data presented in B) and are plotted as a function of XMetS concentration. **D**. CHO-hINSR cells were preincubated for 10 minutes at 37°C with either XMetS or control antibody followed by a 10 minute incubation with increasing concentrations of insulin and phosphorylation of Erk was then determined. The mean of duplicate determinations are shown.

Two major enzymes that are activated via signaling by both isoforms of the INSR are Akt and Erk [2]. Akt is a key mediator of metabolic signaling downstream of the INSR whereas Erk is a key mediator of mitogenic (growth) signaling downstream of the INSR [2]. The influence of XMetS on insulin-mediated Akt and Erk activation in CHO-hINSR cells expressing the B isoform of the INSR was studied. These studies assessed whether the positive modulating effect of XMetS on autophosphorylation of the hINSR B isoform (**Figure 3A** **)** was associated with enhanced downstream intracellular signaling. XMetS induced a greater than 30-fold increase in the sensitivity of insulin-stimulated Akt phosphorylation as observed by a pronounced left shift in the dose response curve (**Figure 4A and 4B**). The EC_50_ for insulin stimulated pAkt activation in the absence of XMetS was 1.1 nM (95% CI: 0.94 to 1.29 nM) and decreased to 0.027 nM (95% CI: 0.022 to 0.032 nM) in the presence of XMetS (**Figure 4A** **)**. At high concentrations, XMetS alone, had only a small (∼10%) direct agonist effect on this function. These data suggested that the potentiation of insulin-mediated INSR activation by XMetS also translated to an enhancement of downstream signaling. The monovalent Fab version of XMetS induced an 8-fold increase in the sensitivity of insulin-stimulated Akt phosphorylation (**Figure 4A**). The EC_50_ for insulin-stimulated Akt phosphorylation in the presence of the Fab form of XMetS was 0.133 (95% CI: 0.103 to 0.173 nM). These data indicated that the modulatory effects of XMetS were independent of antibody valence.

In order to quantitate the potency of XMetS on INSR modulation, the dose response of insulin-dependent pAkt activation was assessed at XMetS concentrations ranging from 0 to 50 nM (**Figure 4B**). The concentration of XMetS required to induce a half-maximal left shift of insulin-dependent Akt phosphorylation dose response curve occurred at 0.25 nM (95% 0.07 CI: to 0.85 nM) (**Figure 4C**). These dose response data were also fit using a mathematical model of allosteric modulation [25] to determine if the antibody was acting to enhance downstream INSR signaling in addition to enhancing insulin binding and INSR activation by autophosphorylation. The calculated cooperativity factor for potentiation of insulin action on the INSR by XMetS was 37 (log αβ = 1.57, 95% CI: 1.41 to 1.73). This cooperativity factor incorporates both positive modulation of insulin binding affinity (α) and positive modulation of signaling efficacy (β) by XMetS. Using the observed enhancement of insulin affinity by XMetS (α) as determined by KinExA™ analysis (18-fold), the enhancement of insulin signaling efficacy (β) in this system was estimated to be approximately 2-fold [35]. The analysis also yielded an estimate of the affinity of XMetS for the INSR in the absence of insulin of 8.5 nM (logK_B_ = 0.93 nM, 95% CI: 0.67 to 1.2 nM), which is similar to the affinity value as determined by KinExA™ analysis for this interaction. These data indicated therefore, that XMetS acted mainly to enhance insulin binding affinity but also significantly increased the efficacy of insulin signaling through the INSR.

XMetS also potentiated insulin dependent Erk1/2 phosphorylation in CHO cells expressing the hINSR, but to a lesser extent than Akt phosphorylation. The EC_50_ for insulin-stimulated pErk1/2 activation in the absence of XMetS was 26.4 nM (95% CI: 13.8 to 50.5 nM) and decreased to 5.5 nM (95% CI: 3.8 to 8.0 nM) in the presence of XMetS (**Figure 4D**). In contrast to pAkt activation, XMetS at high concentrations had no direct agonist effect on pErk1/2 phosphorylation.

#### The effect of XMetS on insulin-mediated glucose uptake and cancer cell proliferation

**Figure 5:**
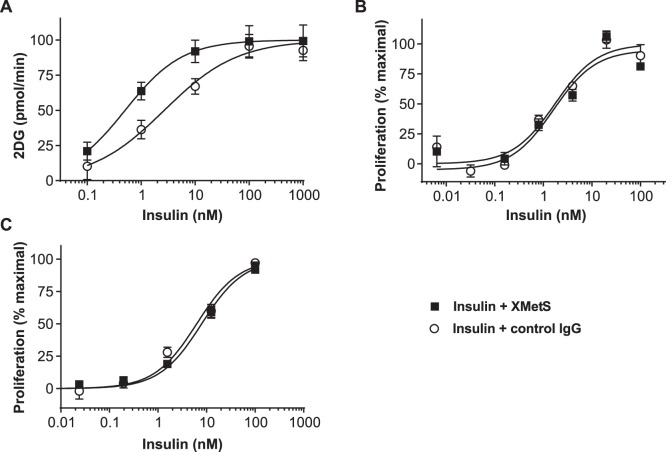
XMetS potentiates insulin-mediated 2-deoxy-D-glucose uptake in L6 muscle cells, but does not enhance the growth of cancer cells. **A.** L6 cells expressing both isoform B of the hINSR and GLUT-4 were preincubated with either XMetS or control antibody for 60 minutes at 37°C followed by a 10 minute incubation with increasing concentrations of insulin. [^3^H]-2-deoxy-D-glucose (2DG) was added and uptake was measured after 20 minutes. **B.** MCF-7 human breast cancer cells were incubated for 48 hours at 37°C with increasing concentrations of insulin in the presence of either XMetS or control antibody, and cell proliferation was determined by CellTiter Glo® assay. **C.** Saos-2 human osteosarcoma cells were incubated for 72 hours at 37°C with increasing concentrations of insulin in the presence of either XMetS or control antibody, and cell proliferation was determined by CellTiter Glo® assay. The mean ± SD from triplicate determinations are shown.

To determine whether XMetS potentiated insulin-stimulated glucose transport in muscle, a major target tissue for insulin, we employed L6 cultured muscle cells that express both hINSR and GLUT-4 [31]. In these cells, XMetS enhanced insulin-stimulated 2-deoxy-D-glucose (2DG) uptake (**Figure 5A** **)**. The EC_50_ for insulin-stimulated 2DG uptake in the absence of XMetS was 2.7 nM (95% CI: 1.4 to 5.3 nM) and decreased to 0.50 nM (95% CI: 0.26 to 0.97 nM) in the presence of XMetS. XMetS alone had little or no effect on this function. Thus, potentiation of insulin binding to the INSR by XMetS resulted in enhanced metabolic signal activation and enhanced glucose uptake.

In addition to its effects on metabolic functions such as glucose transport, insulin can also stimulate the growth and proliferation of cancer cells, a potential concern when either insulin or insulin analogs are employed for therapeutic purposes [36]–[38]. Because XMetS enhanced the binding affinity of the INSR for insulin, we studied the effect of XMetS on insulin-stimulated proliferation of MCF-7 human breast cancer cells. In contrast to the observed impact of XMetS on insulin-stimulated glucose transport, XMetS did not influence the EC_50_ of insulin-stimulated proliferation in these cells, relative to control antibody (insulin plus XMetS EC_50_ = 1.5 nM, 95% CI: 0.4 to 5.3 nM vs. insulin plus control IgG EC_50_ = 1.7 nM, 95% CI: 0.9 to 3.5 nM) **(** **Figure 5B**). XMetS also did not influence the effect of insulin on the proliferation of Saos-2 human osteosarcoma cells (**Figure 5C** **)**. Moreover, XMetS did not influence the effects of IGF-1 on the proliferation of both cancer cell lines (data not shown).

### II. Studies in vivo

#### In vivo studies with XMetS

**Figure 6:**
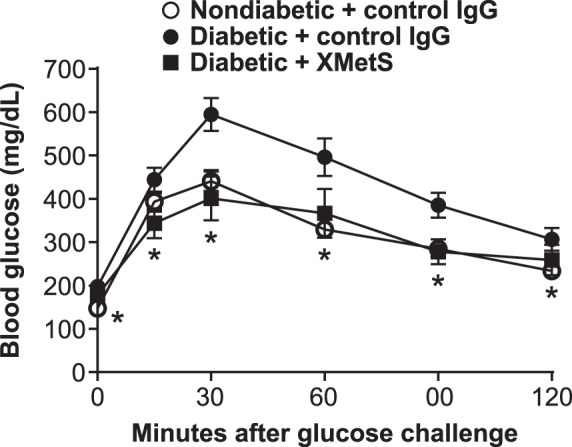
XMetS improves glucose metabolism in diet induced obesity mice. C57BL/6 diet induced obesity mice were treated twice weekly with either control antibody (10 mg/kg) or XMetS (10 mg/kg). Age-matched, lean C57BL/6 mice were treated twice weekly with control antibody (10 mg/kg). After one week of treatment and following a 14-hour overnight fast, a glucose bolus was administered intraperitoneally (1 g/kg) and blood glucose levels were measured for 120 minutes. Mean ± SEM are shown (n = 10 mice/group).

To assess whether the observed positive modulation of the INSR by XMetS *in vitro* translated into an improvement in insulin action *in vivo*, we performed studies in both insulin resistant and diabetic mice. We first assessed the effects of XMetS in diet induced obesity mice, a model of obesity and insulin resistance [32]. After four weeks of treatment, fasting glucose levels in the obese mice treated with control antibody were over 240 mg/dL compared to approximately 160 mg/dL in similarly treated lean mice. Fasting glucose levels in obese mice treated with XMetS for 4 weeks were similar to normal mice treated with control antibody (**Table 1**). Compared to the normal mice, glucose tolerance in obese mice was significantly impaired. Treatment of obese mice with XMetS normalized glucose tolerance (**Figure 6**). In obese mice, fasting insulin levels were twice that of normal mice and fasting C-peptide was also elevated (**Table 1**), reflecting insulin resistance. Treatment of obese mice with XMetS for four weeks lowered both fasting insulin and C-peptide to levels below normal mice. In addition, elevated non-HDL cholesterol levels in obese mice were also significantly improved by XMetS treatment. XMetS treatment did not affect body weight in obese mice, indicating that the improvements in the above parameters elicited by XMetS were not the result of weight reduction (**Table 1**). At no time did any of the mice treated with XMetS manifest symptomatic hypoglycemia.

**Table 1:** Metabolic profile of diet induced obesity mice treated with XMetS and controls.

	Non-diabetic+control IgG	Diabetic+control IgG	Diabetic+XMetS
Body Weight (g)	30.1±1.7*	37.0±3.4	36.8±3.1
Glucose (mg/dL)	163±10*	243±12	189±18*
Total cholesterol (mg/dL)	104±9*	136±12	121±20
Non-HDL cholesterol (mg/dL)	33±8*	63±17	46±14*
Insulin (pg/mL)	338±84*	670±395	209±171*
C-peptide (pM)	224±52*	333±117	112±104*

At the end of the 4-week study with diet induced obesity mice, the animals were fasted 14 hours, weighed and plasma was obtained to determine the above parameters. Mice (n = 10/group) were treated with antibody at 10 mg/kg, twice weekly. Values are shown as mean ± SEM. *p<0.05 vs. diabetic+control IgG.

**Figure 7:**
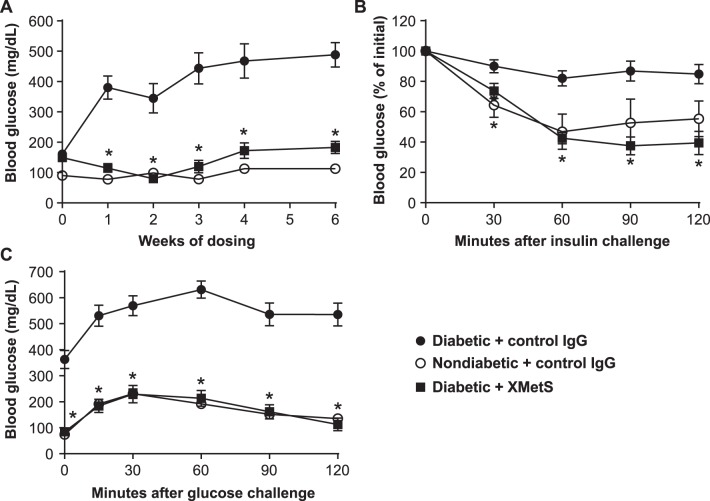
XMetS improves glucose metabolism in multi-low dose, streptozotocin/high-fat diet mice. **A–C.** ICR multi-low dose, streptozotocin/high-fat diet mice were treated twice weekly with either control antibody (10 mg/kg) or XMetS (10 mg/kg). Age-matched non-diabetic ICR mice were treated twice weekly with control antibody (10 mg/kg). **A**. Blood glucose levels were obtained weekly for six weeks following a 14-hour fast. **B**. After five weeks of treatment and following a 4-hour fast, insulin was administered intraperitoneally (0.75 U/kg) and blood glucose levels were obtained for an additional 120 minutes. **C**. After three weeks of treatment and following a 14-hour fast, a glucose bolus was administered intraperitoneally (1 g/kg) and blood glucose levels were measured for 120 minutes. Mean ± SEM are shown (n = 8 mice/group).

We next studied the effects of XMetS in the multi-low dose, streptozotocin/high-fat diet mouse model of diabetes that exhibits both insulin resistance and relative insulinopenia [33]. Diabetic mice treated with control antibody exhibited progressive elevations of fasting glucose levels over six weeks (**Figure 7A** **,** **Table 2**). Fasting hyperglycemia was markedly improved after one week of XMetS treatment and this effect was maintained for the duration of the study. As in diet induced obesity mice, XMetS did not induce symptomatic hypoglycemia.

**Table 2:** Metabolic profile of multi-low dose, streptozotocin/high-fat diet mice treated with XMetS and controls**.**

	Non-diabetic+control IgG	Diabetic+control IgG	Diabetic+XMetS
Body Weight (g)	42.5±3.1*	35.5±3.2	38.4±2.3*
Beta hydroxybutyrate (mmol/L)	0.26±0.05*	4.79±1.8	1.05±0.61*
Glucose (mg/dL)	113±19*	488±40	183±20*
Total cholesterol (mg/dL)	154±17*	175±29	159±31
Non-HDL cholesterol (mg/dL)	34±10*	101±32	63±21*
Insulin (pg/mL)	1006±332*	336±301	137±51*
C-peptide (pM)	204±69*	142±67	67±63*

At the end of the 6-week study with multi-low dose, streptozotocin/high-fat diet mice, the animals were fasted 14 hours, weighed and plasma was obtained to determine the above parameters. Mice (n = 8/group) were treated with antibody at 10 mg/kg, twice weekly. Values are shown as mean ± SEM. *p<0.05 vs. diabetic+control IgG.

To determine whether XMetS improved insulin responsiveness in multi-low dose, streptozotocin/high-fat diet mice, exogenous insulin was administered to mice treated with either XMetS or control antibody. The response to insulin in diabetic mice was significantly compromised relative to that of normal mice, indicating that they were insulin resistant. Treatment of multi-low dose, streptozotocin/high-fat diet mice with XMetS prevented this insulin resistance (**Figure 7B**). In addition, diabetic mice were glucose intolerant. When these mice were treated with XMetS, they displayed markedly improved glucose tolerance with glucose levels similar to those of normal mice (**Figure 7C** **)**.

After six weeks, multi-low dose, streptozotocin/high-fat diet mice had lost significant body weight relative to normal mice as a result of prolonged insulinopenia; XMetS partially prevented this disease-mediated weight loss (**Table 2**). Both C-peptide and insulin were decreased in XMetS-treated diabetic mice relative to diabetic animals treated with control antibody, reflecting increased insulin sensitivity. Beta-hydroxybutyrate levels were markedly elevated in multi-low dose, streptozotocin/high-fat diet mice and these levels were dramatically reduced when the diabetic mice were treated with XMetS. Elevated total cholesterol and non-HDL cholesterol were also reduced in the diabetic animals by XMetS treatment (**Table 2**).

## Discussion

Previously we identified an allosteric anti-INSR antibody, XMetA [18]. We found that XMetA was a direct agonist of the INSR that did not influence insulin binding but activated INSR signaling both in vitro and in vivo. In the present study, we report the identification and characterization of another type of allosteric anti-INSR antibody, XMetS. In contrast to XMetA, XMetS was not a direct agonist of the INSR; rather, XMetS markedly enhanced the affinity of the INSR for insulin. Thus, XMetS was a positive allosteric modulator of the INSR. In accordance with this type of allosteric mechanism we also demonstrated that insulin reciprocally enhanced the binding affinity of XMetS for the INSR as has been shown for other positive allosteric modulators [25]. XMetS potentiated INSR signaling in cultured cells. Moreover, XMetS was active in vivo, improving glucose metabolism in two mouse models of insulin resistance and diabetes.

Others have described monoclonal antibodies to the INSR that were generated using hybridoma technology [4], [39], [40]. In some instances, antibodies were observed to modestly (2-fold or less) increase insulin binding to the INSR [4]. However, neither the in vitro nor in vivo effects of these antibodies on INSR signaling were extensively characterized.

We find that XMetS markedly enhanced insulin binding affinity in cells expressing either the hINSR or the mINSR. Kinetic analysis indicated that XMetS positively modulated insulin binding affinity by decreasing the dissociation rate of insulin from the INSR without affecting the association rate of insulin. These data suggested that XMetS positively modulated the INSR by stabilizing the ligand bound conformation of the receptor resulting in higher affinity for insulin. Both the monovalent Fab and divalent IgG_2_ forms of XMetS acted as positive allosteric modulators of the INSR. This observation suggests that the mechanism by which XMetS stabilized the ligand bound form of the INSR does not require intra- or intermolecular crosslinking if the INSR. Hence, the mechanism for XMetS differs from the proposed mechanism by which insulin activates the INSR via intramolecular crosslinking [41]. It will be important to elucidate the details of XMetS INSR modulation on a structural level. However, such an analysis requires knowledge of the full length ligand activated INSR and the conformation changes associated with activation, both of which have not been fully determined [42], [43].

After insulin binds to the INSR it activates receptor autophosphorylation, which leads in turn to activation of downstream signaling. Similar to its effect on insulin binding affinity, XMetS potentiated insulin-dependent INSR autophosphorylation by 14-fold indicating that the enhancement of insulin binding was associated with improved insulin-mediated INSR activation. The effect of XMetS was specific to the INSR as it did not potentiate activation of the IGF-1R by IGF-1.

A key signaling event triggered by INSR activation is the phosphorylation and activation of the intracellular enzyme Akt [2], which leads to cellular glucose uptake. XMetS markedly enhanced insulin dependent Akt phosphorylation greater than 30-fold in cultured cells expressing the INSR. In accordance with its effect on Akt activation mediated by the INSR, XMetS potentiated insulin-stimulated 2-deoxy-D-glucose uptake in cultured muscle cells expressing the INSR.

In humans, insulin may enhance tumor progression via the INSR, while insulin analogs with INSR dissociation rates slower than insulin may have greater mitogenic activity [44]. XMetS slowed the rate of dissociation of insulin from the INSR. Thus, we studied the effect of XMetS on insulin-stimulated proliferation in MCF-7 breast cancer cells and Saos-2 osteosarcoma cells, two cancer cells lines that are commonly used to determine the mitogenic effects of insulin analogs [37]. We observed that XMetS did not enhance the effect of either insulin or IGF-1 on the proliferation of these cell lines. In the present study we found that the observed potentiation by XMetS on insulin induced Erk activation was less that the potentiation of insulin-stimulated Akt activation by XMetS. Studies have indicated that insulin stimulation of the MAP kinase pathway including enhanced Erk phosphorylation may be associated with mitogenesis [36]. Thus one potential explanation for the lack of effect of XMetS on ligand induced proliferation of cancer cells may be related to the relatively limited effect of XMetS on insulin-dependent Erk activation.

Recently, Vigneri *et. al.* proposed the concept that certain molecules, including monoclonal antibodies, that either activate or modulate metabolic INSR signaling without increasing cell proliferation would be of therapeutic value [38]. We previously reported that XMetA, an INSR agonist antibody, selectively activated metabolic but not mitogenic functions including the proliferation of cancer cells [18]. We now find that XMetS, a positive allosteric modulator of the INSR, also does not agonize or potentiate insulin-mediated cancer cell proliferation. These observations suggest that allosteric antibodies with the unique profiles of either XMetA or XMetS may be of therapeutic value. We therefore studied whether the in vitro ability of XMetS to potentiate the binding of insulin to the INSR and enhance intracellular signaling and glucose transport, were reflected in vivo by enhanced insulin responsiveness and improved glucose metabolism in obese and diabetic animals.

In the diet induced obesity mouse, a model of obese insulin resistance and hyperglycemia, XMetS reduced fasting blood glucose levels and normalized glucose tolerance. Improved glycemic control in these animal models was accompanied by reduced insulin and C-peptide levels, suggesting that XMetS increased insulin sensitivity and lowered the requirement for insulin. This reduction of compensatory hyperinsulinemia caused by XMetS may contribute to the lack of hypoglycemia that was observed in the diseased mice treated with this antibody.

In insulin resistant, insulinopenic multi-low dose, streptozotocin/high-fat diet mice, XMetS also markedly improved fasting hyperglycemia and normalized glucose tolerance. These diabetic mice had a diminished ability to respond to exogenous insulin and were therefore insulin resistant. XMetS restored the ability of insulin to lower glucose levels in these mice, indicating decreased insulin resistance. In concert with this observation, XMetS also lowered insulin and C-peptide values, further indicating that it improved insulin sensitivity. In addition to its beneficial effects on glucose metabolism, XMetS lowered beta hydroxybutyrate levels in multi-low dose, streptozotocin/high-fat diet mice and improved dyslipidemia as reflected by a reduction of elevated non-HDL cholesterol. XMetS also partially ameliorated the disease related weight loss that was observed in these mice.

An INSR mutant has been described (K460E) [45], that exhibits increased affinity for insulin and induces insulin resistance in humans through a proposed mechanism that involves accelerated INSR degradation [46]. Insulin analogues have also been described that exhibit higher affinity for the INSR with enhanced mitogenic activity (e.g. AspB10) ([47]–[49]. These effects are the opposite of those for XMetS which increases insulin sensitivity and does not enhance mitogenic activity. Although direct experimental comparisons with XMetS have not been carried out, the present data suggest that the mechanisms by which these INSR and insulin mutants increase the affinity of insulin binding to the INSR are considerably different than the mechanism(s) employed by XMetS. Further studies on insulin processing and INSR trafficking will be required to elucidate these differential effects.

In humans, the natural history of T2DM has been described in multiple populations [50]–[55]. The insulin resistance of T2DM results in part from obesity and physical inactivity that are now prevalent in many developed and developing countries [56]. Initially, most individuals become insulin resistant due to defects in the INSR signaling pathway [55], [57], [58]. To compensate, the pancreatic beta cell initially produces and secretes additional insulin in an attempt to maintain euglycemia [51], [52], [58], [59]. However, in many instances compensatory insulin secretion eventually diminishes as a result of beta cell dysfunction leading to uncontrolled hyperglycemia. While current treatments for early T2DM patients may initially provide satisfactory regulation of blood glucose, many patients ultimately require exogenous insulin [51], [52], [59]. This progression underscores the need for new therapies that may enhance the ability of endogenous insulin to activate the INSR. Such agents could improve glycemic control, prevent compensatory hyperinsulinemia, and possibly delay or obviate the need for exogenous insulin therapy by preserving beta cell function. The studies described herein demonstrate that an allosteric monoclonal antibody to the INSR, XMetS, positively modulates insulin binding to the INSR resulting in improved glucose metabolism both in vitro and in vivo. Importantly, XMetS does not potentiate insulin-stimulated proliferation of cancer cells. Thus, these studies suggest that this type of allosteric monoclonal antibody could have therapeutic utility for the treatment of T2DM.
